# Long non-coding RNAs: implications in targeted diagnoses, prognosis, and improved therapeutic strategies in human non- and triple-negative breast cancer

**DOI:** 10.1186/s13148-018-0514-z

**Published:** 2018-06-27

**Authors:** Rubén Rodríguez Bautista, Alette Ortega Gómez, Alfredo Hidalgo Miranda, Alejandro Zentella Dehesa, Cynthia Villarreal-Garza, Federico Ávila-Moreno, Oscar Arrieta

**Affiliations:** 1 Thoracic Oncology Unit and Laboratory of Personalized Medicine, Instituto Nacional de Cancerología (INCan), San Fernando #22, Section XVI, Tlalpan, 14080 Mexico City, Mexico; 2 Biomedical Science Doctorate Program, National Autonomous University of Mexico, Mexico City, Mexico; 3 Cancer Genomics Laboratory, INMEGEN, Mexico City, Mexico; 4 Biochemistry Department, Instituto Nacional de Ciencias Médicas y Nutrición Salvador Zubirán, Mexico D.F, Mexico; 5 Breast Oncology Department, National Cancer Institute of Mexico, Mexico City, Mexico; 6 Lung Diseases And Cancer Epigenomics Laboratory, Biomedicine Research Unit (UBIMED), Facultad de Estudios Superiores (FES) Iztacala, National University Autonomous of México (UNAM), Mexico City, Mexico; 7 Research Unit, National Institute of Respiratory Diseases (INER) “Ismael Cosío Villegas”, Mexico City, Mexico

**Keywords:** Breast cancer, Triple negative, Biomarkers, lncRNA

## Abstract

Triple-negative breast cancer (TNBC) has been clinically difficult to manage because of tumor aggressiveness, cellular and histological heterogeneity, and molecular mechanisms’ complexity. All this in turn leads us to evaluate that tumor biological behavior is not yet fully understood. Additionally, the heterogeneity of tumor cells represents a great biomedicine challenge in terms of the complex molecular—genetical-transcriptional and epigenetical—mechanisms, which have not been fully elucidated on human solid tumors.

Recently, human breast cancer, but specifically TNBC is under basic and clinical-oncology research in the discovery of new molecular biomarkers and/or therapeutic targets to improve treatment responses, as well as for seeking algorithms for patient stratification, seeking a positive impact in clinical-oncology outcomes and life quality on breast cancer patients.

In this sense, important knowledge is emerging regarding several cancer molecular aberrations, including higher genetic mutational rates, LOH, CNV, chromosomal, and epigenetic alterations, as well as transcriptome aberrations in terms of the total gene-coding ribonucleic acids (RNAs), known as mRNAs, as well as non-coding RNA (ncRNA) sequences. In this regard, novel investigation fields have included microRNAs (miRNAs), as well as long ncRNAs (lncRNAs), which have been importantly related and are likely involved in the induction, promotion, progression, and/or clinical therapeutic response trackers of TNBC. Based on this, in general terms according with the five functional archetype classification, the lncRNAs may be involved in the regulation of several molecular mechanisms which include genetic expression, epigenetic, transcriptional, and/or post-transcriptional mechanisms, which are nowadays not totally understood.

Here, we have reviewed the main dis-regulated and functionally non- and well-characterized lncRNAs and their likely involvement, from a molecular enrichment and mechanistic point of view, as tumor biomarkers for breast cancer and its specific histological subtype, TNBC. In reference to the abovementioned, it has been described that some lncRNA expression profiles correspond or are associated with the TNBC histological subtype, potentially granting their use for TNBC malignant progression, diagnosis, tumor clinical stage, and likely therapy. Based on this, lncRNAs have been proposed as potential biomarkers which might represent potential predictive tools in the differentiated breast carcinomas versus TNBC malignant disease. Finally, elucidation of the specific or multi-functional archetypal of lncRNAs in breast cancer and TNBC could be fundamental, as these molecular intermediary-regulator “lncRNAs” are widely involved in the genome expression, epigenome regulation, and transcriptional and post-transcriptional tumor biology, which in turn will probably represent a new prospect in clinical and/or therapeutic molecular targets for the oncological management of breast carcinomas in general and also for TNBC patients.

## Background

The most recent worldwide cancer statistics estimated a total of 2.4 million new cases and 533,000 deaths due to breast cancer (BC) in 2015, thus making it the fifth leading cause of cancer years of life lost between 2005 and 2015 for both sexes. For women specifically, one in 14 will develop BC between birth and 79 years of age, becoming the leading cause of cancer death for women worldwide [1]. In 2017, 252,710 new breast malignant clinical cases and 40,610 BC deaths were expected to occur in the USA [2].

In addition to the known histopathological classification (tumor cell differentiation status) and TNM (tumor size, lymph node involvement, and metastasis) stage, BC has also been classified on the basis of protein and genetic expression status [3]. In this regard, Perou et al. have defined at least five genetically distinct subtypes with different molecular significance on inter-tumor subtypes [4], but also by intra-tumor heterogeneity in BC subtypes [5]. Based on the above, intra-tumor heterogeneity has been proposed as having striking morphological, genetic, and behavioral variability explained in part by the cancer stem cells population presence, clonal evolution, and malignancy capacity [6, 7]. Additionally in BC, other molecular-genetic alterations exist based on well-known non-coding RNAs (ncRNAs), including micro-ncRNAs (microRNAs) and long-ncRNAs (lncRNAs), both of which have been identified and histopathologically associated to BCs, as triple-negative BC (TNBC) [8, 9], but particularly some lncRNAs, in others as LOC339535 also named LINK-A, have functionally been associated to TNBC malignancy with poorer prognosis and progression-free survival in TNBC patients [10], as well as for HOTAIR, which has been involved in promoting or increasing malignancy in TNBC patients, compared with non-TNBC patients, probably representing a new target therapy in TNBC [11].

TNBC as a heterogeneous group of breast tumors has been characterized by the lack of expression of hormone receptors, namely estrogen receptor alpha (ER-α) and progesterone receptor (PR), with a low expression of receptor tyrosine kinase ErbB2 (also known as HER2/neu). In TNBC, additional molecular-genetic features have been identified including BRCA1/2 mutation frequency (11.2–20.0%) [12–15], which may be higher in approximately 20–40%, according to the ethnic origin [16], furthermore including additional molecular deficiencies, in others as some frequent somatic mutations on TP53 (62%) and PIK3CA (10%) [12, 13].

In addition to the mutation status, TNBC tumors also display alterations on the genetic copy number variations, genetic expression levels, and patterns, which have been associated with basal-like tumors including a high proportion of the basal histological BC subgroup (70–80%) [17]. Besides TNBCs exhibiting poor survival rates due to their highly aggressive and metastatic capacities, they are associated with higher recurrence behavior in local and distant lymph nodes and have higher proliferative rates [18–20], probably explained in part by the genetic-molecular aberrations.

Approximately, on average, 12 to 24% of women diagnosed with BC correspond to the TNBC subtype. TNBC represents a subgroup of particular interest, since it generally affects young women and tends to have a poor response to standard chemotherapy [21–23].

In TNBC, the US Food and Drug Administration (FDA) and the European Medicines Agency (EMA) have not yet approved a specific targeted agent for clinical treatment in the adjuvant, neoadjuvant, or metastatic settings. Currently, radiotherapy and a combination of chemotherapeutic agents like anthracyclines, alkylating agents, taxanes, or platinum salts are used for treating TNBC patients [24]. Thus, efficient targeted therapeutic regimens are urgently needed in TNBC for clinical management, since currently these patients have low rates of disease-free survival (DFS), overall survival (OS), and 5-year survival, in addition to a low survival 12–18 months after distant recurrence [25, 26].

TNBC tumors have been characterized by high levels of genetic instability, with a median of 1.7 (range 0.16–5.23) mutations/Mb [27, 28], and feature complex patterns of copy number gains and losses throughout the genome [29]. Epigenetically TNBCs are characterized by extensive hypomethylation, which leads to increased genome-wide instability [30]. Recently, Mathe et al. have shown that the changes in the epigenome, based on DNA methylation levels, are associated with tumor progression in TNBC [31–33].

Recent reports have shown that lncRNAs are involved in almost all human biological processes including transcriptional regulation or interference, telomere maintenance, epigenetic mechanisms modulation, imprinting, post-transcriptional and translational control, structural organization, cellular differentiation, embryo development, and pathological dysfunctions as well as non-malignant diseases, using redundant DNA, RNA, and/or protein-binding mechanisms, according to particular cases [34–36]. As it occurs for other malignant diseases, lncRNAs have been involved in the tumorigenic promotion and progression processes leading to BC development and prognosis [37].

Liu et al. proposed a TNBC classification based on mRNA coding genes and lncRNA expression profiles. This new classification could offer a more robust data matrix to establish a molecular stratification bioinformatics-algorithm that clarifies knowledge of molecular subtypes and establishes subtype-specific targets [38]. Additionally, genome-wide association studies on cancer have revealed that more than 80% of cancer-associated single-nucleotide polymorphisms occur in non-coding genetic regions. This suggests that a significant fraction of the genetic etiology of BC could be related to lncRNA expression profile and functionality [39]. Also, research involving genetic sequence control (promoters vs. enhancers) is necessary, in order to explain why, how, and where lncRNAs are expressed in human homeostasis as well as during a pathologic process [40].

**Fig. 1:**
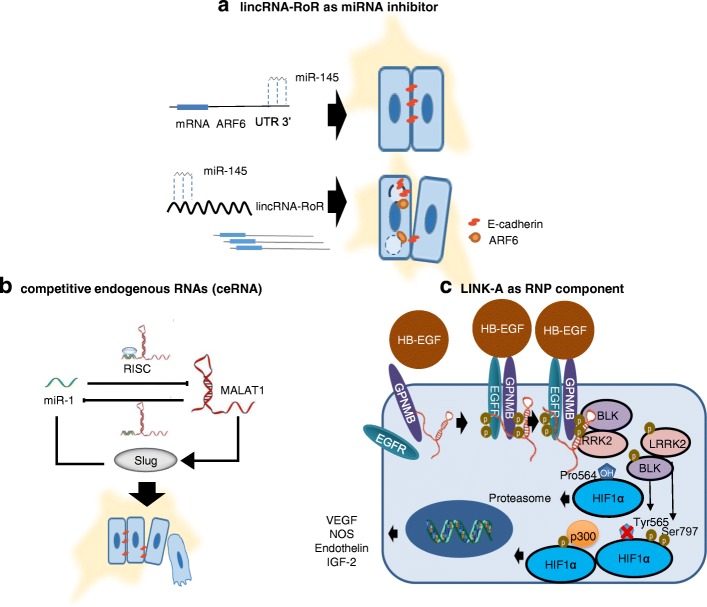
Proposed five functional archetypes for the lncRNA mechanisms. 1. Decoys: lncRNAs can titrate away transcription factors and other proteins away from chromatin, or titrate the protein factors into nuclear subdomains. 2. Signals: lncRNAs expression can faithfully reflect the combinatorial actions of transcription factors (colored ovals) or signaling pathways to indicate gene regulation by space and time. 3. Guides: lncRNAs may recruit chromatin-modifying enzymes to gene-promoter targets, either in *Cis* (near the genetic region of the lncRNA transcription) or in *Trans* into distant target genes. 4. Scaffolds: lncRNAs may bring together multiple proteins to conform ribonucleoprotein complexes. The lncRNA-RNP may act on chromatin as illustrated to affect histone code modifications. In other instances, the lncRNA scaffold is structural and stabilizes nuclear structures or signaling complexes 5. Sponge: lncRNAs that by complementarity of bases succeed in matching or sequestering sequences of small non-coding RNAs, such as miRNAs, are controlling bioavailability of miRNAs, vs. lncRNAs themselves, with the functional biological repercussions at cellular or physiological level. RNA-induced silencing complex RISC

In recent years, therapeutic strategies for TNBC have recorded a high number of failures in the development of chemical agents, due to the fact that recently it has been proposed to include molecular wide studies to identify additional potential biomarkers, as well as genetic-epigenetic targets, probably involved. In this regard, epigenetic targeted pathways have widely been proposed as pharmacological strategies, among these histone deacetylase inhibitors (HDACis) alone and/or in combination strategies have promising activity in TNBC-targeted treatments. Therefore, future research should be focusing on the personalized approach, which will benefit more from each kind of epigenetic agents, including panobinostat, vorinostat, and entinostat [41]. In addition, by identifying reliable treatment biomarkers, such as lncRNAs, which are implicated in epigenetic mechanisms through the recruitment of the chromatin modification complexes, in other proteins based on the polycomb repressor complex-2 (PRC2) and/or LSD1/CoREST (REST co-repressor) REST complexes, involved in the histone repressor (H3K27me3) versus activation (H3K4me2/3) code, as it has been previously described for the lncRNA HOTAIR, suggesting a scaffold functionality archetype [42], as lncRNAs have functionally been classified by five archetypes (described in Fig. 1).

In brief, the present review summarizes the current knowledge regarding lncRNA expression patterns and probable functional association with their role in BC biology and as expressed molecular biomarkers, potentially involved as therapeutic targets. In this regard, we aim to generate a systematic and deep understanding of tumor biology of TNBC, so clinicians will, in the near future, be able to offer tailored treatments in accord to the lncRNA stratification and/or specific lncRNA expression patterns in potentially different patient subgroups in BC and TNBC.

## Targeted therapy efficacy in the TNBC treatment: probable lncRNAs involved

Due to the lack of knowledge on molecular targets, chemotherapy is the only available systemic treatment for TNBC and therefore adjuvant chemotherapy is recommended for TNBC operable tumors with stages I–III [43, 44]. However, systemic therapy before surgery, neoadjuvant chemotherapy (NAC), is the most appropriate approach for patients with locally advanced BC with the objectives to improve surgical options (resectability and breast conservation techniques), determine in vivo tumor sensitivity to treatment, and improve long-term survival outcomes with the pathological complete response (pCR) as an informative biomarker of those parameters [45–48]. Even with what is deemed as a poor overall survival, it is evident that subsets of TNBC patients respond better to standard care using chemotherapy combinations, and when pCR after NAC is achieved; excellent long-term survival is expected [49]. Nevertheless, a substantial proportion (30–40%) of patients with early-stage TNBC develop metastatic disease [50].

In this sense, the triple-negative paradox on TNBC patients is mainly driven by a subgroup of cells on the bulk tumor with residual disease after NAC [50]. For this reason, the search for new biomarkers would allow the prediction of a group of TNBC patients who would better respond to standard chemotherapy, thus eliminating the need to administer unnecessary, highly toxic, and costly chemotherapy treatments in patients who might benefit from more personalized treatments.

Researchers have proposed new targeted therapies based on results from clinical trials in an attempt to improve the outcome of TNBC patients. Retrospective analyses and previous trials have shown striking pCR rates in patients with high *BRCA1* mutation rates (between 72 and 90%) with a single neoadjuvant treatment using DNA crosslinking platinum salts (e.g., cisplatinum) [51, 52]. For example, TNBC patients with positive *BRCA* mutations treated with carboplatin have better response rates compared to those treated with docetaxel monotherapy [53]. Other studies have evaluated poly (ADP-Ribose) polymerase (PARP) inhibitors either alone or in combination with cytotoxic treatment. However, response to these schemes is limited to patients with BRCA-mutated BC [54, 55].

Likewise, hyper-activation of the PI3K/AKT signaling pathway is associated to oncogenic alterations in TNBC, occurring in approximately 10% of patients [56]. Activating PIK3CA mutations are the most frequent mutations in TNBC. Other alterations in this pathway include loss of tumor suppressor phosphatases inositol polyphosphate 4-phosphatase type II (INPP4B), loss of phosphatase and tensin homolog (PTEN), AKT amplification, and AKT3 translocation [57–59]. In this regard, several studies have demonstrated the benefits of using serine/threonine kinase AKT inhibitors like ipatasertib in TNBC [37, 38, 60].

On the other hand, growth factor receptors are overexpressed in TNBC, including epidermal growth factor receptor (EGFR) and vascular endothelial growth factor receptor (VEGFR) [61–66]. Multiple signaling pathways, such as PI3K/AKT, mitogen-activated protein kinase (MAPK), and Wnt/β-catenin are activated by EGFR and, in turn, enhance proliferation, survival, invasion, and metastasis of cancer cells [67]. Expression of EGFR is frequently associated with TNBC and has been viewed as a promising therapeutic target. Unfortunately, the therapeutic efficacy of EGFR-targeting agents in BC has been disappointing [68, 69]. A recent report showed that combined treatment with lapatinib, a dual inhibitor of EGFR and ErbB2/HER2, and imatinib, a c-ABL inhibitor, resulted in synergistic growth inhibition in a panel of EGFR/ErbB2-expressing BC cells, including the TNBC cell line MDAMB-468 [70].

Recently, studies on fibroblast growth factor receptor (FGFR) have shown that 9% of TNBC with FGFR1 (4%) and FGFR2 amplifıcations, treated with FGFR blockers like lucitanib (FGFR1-amplifıcation) and JNJ-42756493 (FGFR translocation or FGFR activating mutation), provide clinical benefits [51, 71–73]. Approximately 10 to 15% of TNBC express the androgen receptor (AR), and several studies have reported pathological response benefits when targeting this receptor. Bicalutamide, enzalutamide, and orteronel are all oral non-steroidal anti-AR, on that the most recent clinical trials for TNBC are shown in Table 1 [72, 74–76].

**Table 1:** The most recent clinical trials in TNBC patients in the search of therapeutic biomarkers for advanced disease

Clinical trial	Phase	Study groups	Indications	Therapeutic target
NCT02623972	II	Eribulin (Halaven®) by IV, for 4 cycles; followed by AC by IV, for 4 cycles	Advanced TNBC	Inhibitor of microtubule dynamics
NCT02120469	I	Everolimus (Afinitor®) by OA daily; eribulin mesylate (Halaven®) by IV twice every month	Metastatic TNBC	mTOR inhibitor
NCT02672475	I	Paclitaxel (Taxol®), by IV, weekly, 3 weeks on, 1 week off; Galunisertib (LY2157299), by OA, twice daily, 3 weeks on, 1 week off	Metastatic TNBC	Inhibitor of the TGF-β receptor I kinase
NCT02632071	I	ACY-1215 (ricolinostat), by OA, daily for 3 weeks on, 1 week off; nab-paclitaxel (Abraxane®), by IV, weekly for 3 weeks on, 1 week off	Advanced TNBC	HDAC6 blocker
NCT02393794	I–II	Romidepsin (Istodax®) by IV, twice every 3 weeks; cisplatin (Platinol®), by IV, every 3 weeks	TNBC or BRCA1 or BRCA2 mutation-associated locally recurrent or metastatic BC	HDAC inhibitor
NCT02425891	III	Experimental group: MPDL3280a (atezolizumab), by IV; nab-paclitaxel (Abraxane®), by IV Control group: placebo; nab-paclitaxel (Abraxane®), by IV	Locally advanced or metastatic TNBC	Anti-PD-L1
NCT02366949	I	Experimental group: BAY1217389, by OA, twice daily; paclitaxel (Taxol®) by IV, weekly Control group: paclitaxel (Taxol®) by IV, weekly	Advanced TNBC	MPS1
NCT02309177	I	Group 1 nab-paclitaxel, by IV, weekly for 3 weeks every month; nivolumab (Opdivo®), by IV, every 2 weeks, starting at 3 months; group 2 nab-paclitaxel, by IV, once every 3 weeks 28/02/2017 nivolumab (Opdivo®), by IV, every 3 weeks, starting at 3 months	HER2−, recurrent metastatic TNBC	Anti-PD-1
NCT02595320	II	Group 1: 1500 mg capacitabine (Xeloda®), by OA, twice daily, 1 week on, 1 week off; group 2: 1250 mg capacitabine (Xeloda®), by OA, twice daily, 2 weeks on, 1 week off	Metastatic TNBC	Alkylating agent; tumor-selective and tumor-activated cytotoxic agent
NCT02897375	I	Group 1; palbociclib (Ibrance®), by OA, daily (3 weeks on, 1 week off); cisplatin (Platinol®), by IV, once, monthly; group 2: palbociclib (Ibrance®), by OA, daily (3 weeks on, 1 week off); carboplatin (Paraplatin®), by IV, once, monthly	ER +, HER2− metastatic BC, advanced BC	CDK inhibitor
NCT00978250	II	FdCyd (5-fluoro-2′-deoxcytidine) and THU (tetrahydrouridine) by IV for 5 days per week for 2 weeks, followed by 2 weeks of no treatment	Advanced BC	FdCyd, a fluoropyrimidine nucleoside DNMT inhibitor, and THU,THU does not have any anticancer effects, but it can help keep the other drug
NCT02046421	I	Mifepristone by OA on days 0, 1, 7, and 8; carboplatin (Paraplatin) and gemcitabine hydrochloride (Gemzar) by IV on days 1 and 8	Advanced BC	GR antagonist
NCT02752685	II	Pembrolizumab (Keytruda®), by IV, every 3 weeks; nab-paclitaxel (Taxotere®), by IV, weekly, 2 weeks on 1 week off	HER2− metastatic BC	Anti PD-1
NCT02915744	III	Group 1: NKTR-102, by IV, once every 3 weeks, ongoing; group 2 treatment of physician’s choice (eribulin/Halaven®, ixabepilone/Ixempra®, vinorelbine/Navelbine®, gemcitabine/Gemzar®, paclitaxel/Taxol®, docetaxel/Taxotere® or nab-paclitaxel/Abraxane®), by IV	Metastatic BC with brain metastases	Topoisomerase I inhibitor
NCT02929576	III	Group 1: Xtandi and Taxol, enzalutamide (Xtandi®), by OA, daily, ongoing, paclitaxel (Taxol®), by IV, weekly for 16 weeks; group 2: placebo and Taxol, placebo, by OA, daily, ongoing, paclitaxel (Taxol®), by IV, weekly for 16 weeks; group 3: Xtandi followed by Taxol, enzalutamide (Xtandi), by OA, daily, ongoing, followed by paclitaxel (Taxol®), by IV, weekly for 16 weeks	Advanced TNBC	Synthetic non-steroidal antiandrogen
NCT02163694	III	Group 1: experimental: veliparib by OA, on days 2 through 5, carboplatin (Paraplatin®) and paclitaxel (Taxol®) by IV, once every 3 weeks; group 2: control: placebo by OA on days 2 through 5, carboplatin and paclitaxel by IV, once every 3 weeks	Advanced HER2− BCr with BRCA1 or BRCA2 mutation	PARP inhibitor
NCT02187991	II	Group 1: paclitaxel (Taxol®), by IV, 3 times a month, ongoing; Group 2: paclitaxel (Taxol®), by IV, 3 times a month, ongoing, alisertib, by OA, 3 times a week, ongoing	ER+/HER2− or advanced TNBC	Aurora A kinase inhibitor
NCT01990352	II	Doxil® (pegylated liposomal doxorubicin hydrochloride), by IV, every 3 weeks	Metastatic TNBC	Liposoma
NCT01999738	I	EC1456 by injection, twice a week on weeks 1 and 2 of every month	Metastatic TNBC	Injectable targeted SMDC consisting of folate (vitamin B9; a folate receptors agonist) covalently linked to the potent mitotic poison and cytotoxic agent, tubulysin B hydrazide (Tub-B-H, a tubulin polymerization inhibitor)
NCT02950064	I	BTP-114, by IV, once every 3 weeks, ongoing	Advanced TNBC with a BRCA 1/2 mutation	Albumin-binding cisplatin prodrug
NCT01802970	I	Anakinra (Kineret®) alone, by OA, for 2 weeks followed by: anakinra (Kineret®), by OA, nab-paclitaxel (Abraxane®) by IV weekly, for a maximum of 6 months	Advanced BC	IL-1 receptor antagonist, an anti-inflammatory
NCT02000882	II	BKM120 by OA daily; capecitabine by OA twice a day, for 2 weeks on, 1 week off, ongoing	TNBC with brain metastases	PI3K inhibitor
NCT02379247	I–II	BYL719, by OA, daily; nab-paclitaxel (Abraxane®) by IV, weekly for 3 out of every 4 weeks	Advanced HER2-negative BC	PI3K inhibitor
NCT02624700	II	Pemetrexed, by IV, every 2 weeks; sorafenib, by OA, twice daily for 5 days	Recurrent or metastatic TNBC	Small inhibitor of several tyrosine protein kinases, such as VEGFR, PDGFR, and Raf family kinases (more avidly C-Raf than B-Raf)
NCT02978716	II	Group 1: chemotherapy only, gemcitabine (Gemzar®) and carboplatin (Paraplatin®), by IV, on days 1 and 8, ongoing; group 2: trilaciclib and chemotherapy, trilaciclib (G1T28), by IV, on days 1 and 8, ongoing, gemcitabine (Gemzar®) and carboplatin (Paraplatin®), by IV, on days 1 and 8, ongoing; group 3: trilaciclib and chemotherapy, trilaciclib (G1T28), by IV, on days 1, 2, 8, 9, ongoing, gemcitabine (Gemzar®) and carboplatin (Paraplatin®), by IV, on days 2 and 9, ongoing	Recurrent or metastatic TNBC	CDK4/6 inhibitor
NCT02753595	I–I	Eribulin mesylate (Halaven®), by IV, weekly (2 weeks on, 1 week off); PEGPH20, by IV, weekly (2 weeks on 1 week off)	HER2− metastatic BC	Pegylated recombinant human PH20 degrades hyaluronic acid (HA) coating tumor cells
NCT02762981	I–II	CORT125134, by OA, daily, ongoing; nab-paclitaxel (Abraxane®), by IV, weekly (3 weeks on 1 week off), ongoing	Advanced BC	GR antagonist

Intravenous IV, oral administration OA, AC Adriamycin® and Cytoxan®, mTOR mammalian target of rapamycin, HDAC6 histone deacetylase 6, BRCA1/2 breast cancer 1/2, PD-L1 programmed cell death ligand-1, MPS1 serine/threonine kinase monopolar spindle 1, HER2 human epidermal growth factor receptor 2, PD-1 programmed cell death receptor, ES+ estrogen receptor positive, CDK cyclin-dependent kinase, FdCyd 5-fluoro-2′-deoxcytidine, THU tetrahydrouridine, DNMT DNA methyltransferase, GR glucocorticoid receptor, PARP poly (ADP-ribose) polymerase, SMDC small molecule drug conjugate, IL1 Interleukin-1, PI3K phosphatidylinositol 3-kinase, VEGF vascular endothelial growth factor, PDGF-R platelet-derived growth factor receptors

As previously described, TNBC is a heterogeneous disease, and even though a high number of targeted therapies have been clinically tested, this has not yet translated into a substantial clinical benefit for TNBC patients. Hence, it is necessary to identify highly sensible biomarkers for a better stratification and treatment of these patients. Recently, long non-coding RNAs (lncRNAs) have been reported to drive many important cancer phenotypes through their interactions with other cellular macromolecules [77, 78]. To date, it has been strongly proposed that a deeper functional understanding of lncRNAs will provide novel insights into the molecular mechanism of cancer. As such, lncRNAs are likely to serve as the basis for many clinical applications in oncology [79], like potential biomarkers for diagnosis or therapy targets for clinical treatment of TNBC, as we discuss next.

## lncRNAs: molecular mechanisms and potential therapeutic functionality

Protein-coding gene sequences represent a minority (less than 2%) of the human genome sequences; in contrast, the majority are represented by protein non-coding genome sequences, such as non-coding RNAs (ncRNAs) [80]. The ncRNAs can be divided into two categories: *house-keeping* ncRNAs (tRNA, rRNA, etc.) and *regulatory* ncRNAs (miRNA, lncRNA, piRNA, etc.) [81]. LncRNAs are regulatory ncRNAs with at least 200 nucleotides long (nt) that do not encode any protein [82].

Based on the genomic location sites of the lncRNA transcripts and their neighboring relation with the protein-coding genes, lncRNAs can be divided into five categories: (1) sense lncRNAs, which overlap one or more exons of transcripts on the same strand; (2) antisense lncRNAs, which overlap one or more exons of another transcript on the opposite strand; (3) bidirectional lncRNAs, which are located on the opposite strand from the neighboring exon whose transcription orientation has been identified at less than 1000 base pairs; (4) intronic lncRNAs, which are structurally located within another intron of another transcript; and (5) intergenic lncRNAs, which interact within the genomic interval between two genes [83]. In addition, many known lncRNAs have been identified *intracellularly* either within the cytosol and/or between the nuclear and cytoplasm compartments [84]. According to recent studies, the human transcriptome contains up to 16,000 lncRNAs, frequently spliced and polyadenylated, whose non-coding genes are mainly transcribed by the RNA polymerase II [85].

Raised evidence supports that lncRNAs have potential, diverse, and deep functional roles at the nucleus level, which include acting as a positive (activation) mechanism of the transcriptional regulation, as well as their involvement in the inactivation of epigenetic mechanisms (Eg., X-chromosome inactivation), heterochromatin conformation, telomere maintenance, and pluripotency capacity modulation and also have been seen to be involved in cancer development [86–88].

It has become increasingly important to link clinical correlation studies and experimental evidences, which has suggested that lncRNAs contribute to tumor promoting, progression, and metastasis for different malignant diseases through several cellular processes, ranging from transcriptional (cis/trans) and post-transcriptional regulation mechanisms in cell cycle distribution control and cell differentiation to epigenetic modification mechanisms [89–92]. LncRNAs modulate gene transcription by rearranging chromatin via chromosomal looping and by affecting the binding of transcription factors. LncRNAs also affect miRNA functions by controlling pre-mRNA splicing or as miRNA sponges. Recently, accumulating evidence indicates that there is aberrant expression of lncRNAs in many cancer types [93]. An increasing number of studies have demonstrated that a number of lncRNAs are not transcriptional noise, but have important functions, such as regulating gene expression at various molecular levels, including RNA, miRNA, DNA, and proteins, playing important roles in RNA translation and cytoplasmic protein trafficking [94]. Few studies like Yang et al. have focused on how lncRNA genes themselves are regulated by different transcripts activating regulatory regions of lncRNAs [95].

Other studies have indicated that altered expression levels of lncRNAs are associated with human diseases, including BC. Examples include the lncRNAs H19, HOTAIR (HOX transcript antisense RNA), and UCA1 (urothelial cancer associated 1, non-protein coding), which silence tumor suppressor genes. Likewise, lincRNA-p21 mediates global gene repression in the p53 response, while GAS5 plays a tumor suppressor role [96–100]. Another specific example is CYTOR (cytoskeleton regulator RNA), which plays a role in BC, regulating genes involved in the EGFR/mammalian target of the rapamycin pathway and is required for cell proliferation, cell migration, and cytoskeleton organization [101]. Other lncRNAs have been associated with drug resistance to standard BC treatment. Examples include ARA-lncRNA (adriamycin resistance associated), which provided novel insights into adriamycin resistance. Breast cancer antiestrogen resistance 4 (BCAR4) is related to tamoxifen resistance and could also sensitize BC cells to lapatinib. Lastly, CCAT2 (colon cancer-associated transcript 2) may be downregulated by chemotherapy with 5-FU, blocking different pathways involved with cell migration [102–105]. Other potential targeted lncRNA for breast cancer treatment include SPRY4-IT1 and PANDAR [8, 100]. However, recent studies have revealed that the dysregulation of lncRNAs that are known to be associated with human disease is often due to the aberrant expression of transcription factor inducers that could initiate oncogenic mechanisms by feedback complexes [8].

Recently, Lv et al. found lncRNAs as ANRIL, HIF1A-AS2, and UCA1 expression was significantly increased in plasma of patients with TNBC [106], suggesting their use as TNBC-specific diagnostic biomarkers and/or molecular prognostic predictors [106, 107].

## LncRNAs in TNBC: biology and their potential therapeutic for clinical oncology

Non-coding sequences have a crucial participation for genetic expression regulation or modulation of several genes implicated in BC. However, it remains to be described a total pattern or profile expression of the long non-coding RNAs for the TNBC subgroup that could be implicated in the invasiveness malignity of these tumors.

First at all, Shen et al. identified 1758 lncRNAs and 1254 mRNAs with significant expression differences in TNBC vs. normal adjacent tissue based in microarray analysis [108]; subsequently, Yang et al. and other researcher groups have been working on the identification and validation of the differential expression of lncRNAs by RNA massive sequencing methods (RNA-seq) [9, 79], as well as, more recently single-cell RNA sequencing (scRNASeq) that allows the quantification of transcript expression profiles for individual cells in a cellular population of solid tumor [107, 109]. Following, on 2015, Chen et al. discovered and validated a set of novel aberrant lncRNA profile expressed in TNBC, suggesting that deregulated lncRNA pattern may play a role in the developmental and progression of TNBC (Table 2).

**Table 2:** Main lncRNAs associated with triple-negative breast cancer

Author	lncRNA	Alteration in TNBC	Function/characteristics
Augoff et al. 2012 [127]	LOC554202	Upregulated	MIR31 host gene, regulates proliferation and migration in breast cancer cells and promotes hypermethylation of miR31 in TNBC
Chen et al. 2015 [110]	LINC00993	Upregulated	Associated with the expression of the estrogen receptor and the expression levels of ANKRD30A
	TCONS_l2_00002973	Upregulated	Associated with the expression of the estrogen receptor.
	TCONS_l2_00003939	Upregulated	Associated with the expression of the estrogen receptor.
	TCONS_l2_00002974	Upregulated	Associated with the expression of the estrogen receptor.
Eades et al. 2015 [118]	lincRNA-RoR	Upregulated	Prevents the core TFs from miRNA-mediated suppression in self-renewing human SC
Wang et al. 2015 [119]	HOTAIR	Upregulated	Regulates chromatin state. It is required for gene silencing of the HOXD locus by PRC2, highly expressed in metastatic breast cancers. High levels of expression in primary breast tumors are a significant predictor of subsequent metastasis and death
	MALAT1	Upregulated	Alternative splicing, nuclear organization, epigenetic modulating of gene expression, and a number of evidences indicate that MALAT1 also closely relate to various pathological processes, ranging from diabetes complications to cancer. It regulates the expression of metastasis-associated genes, with proliferation, motility, and apoptosis evasion
Lin et al. 2016 [10]	LINK-A (also known as LOC339535 and NR_015407)	Upregulated	Is an RNA of binding to kinases that phosphorylate HIF 1 alpha in different sites to the canonical ones in human cancer
	RMST	Downregulated	Tumor suppressor
Yang et al. 2016 [79]	LINC01234	Up/downregulated	Oncogene/tumor suppressor
Koduru et al. 2017 [9]	lnc-DNAJC16	Upregulated	Belonging to the DnaJ heat shock protein family, functions in protein translation, translocation and degradation
	lnc-PURA	Upregulated	It is a sequence-specific, multi-functional single-stranded-DNA/RNA-binding protein and RNA-binding protein which can act as a transcriptional activator and repressor

lncRNA, long non-coding RNA; ANKRD30A, Ankyrin repeat domain 30A; TFs, transcription factors; miRNA, microRNA; PRC2, polycomb repressive complex 2; HIF-1α, hypoxia-inducible factor 1 alpha; lincRNA-RoR, long intergenic non-protein coding RNA, regulator of reprogramming; HOTAIR, HOX transcript antisense RNA; MALAT1, metastasis-associated lung adenocarcinoma transcript 1; LINK-A, long intergenic noncoding RNA for kinase activation; RMST, rhabdomyosarcoma 2-associated transcript

**Fig. 2:**
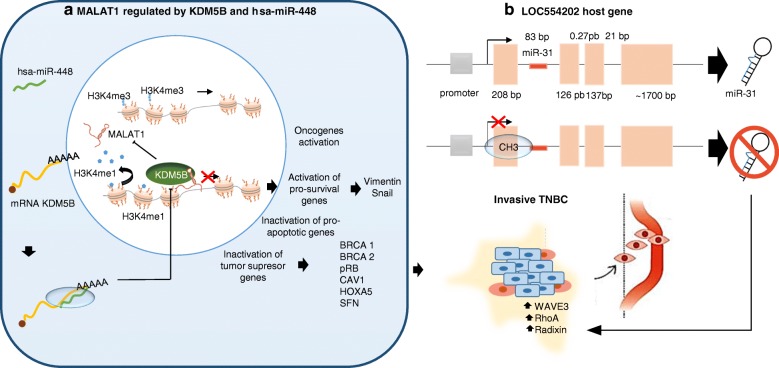
A molecular mechanism model for lncRNAs involved in the tumorigenesis of human TNBC. **a** lincRNA-RoR as a miR-145 inhibitor (oncogene miRNA). **b** MALAT1 as a competitive endogenous RNA of miR-1 (tumor suppressor miRNA). **c** LINK-A as a component of ribonucleoprotein complexes, example shows the regulations of HIF1α pathway. ARF6 ADP-ribosylation factor 6, UTR 3′ untranslated region 3, RISC RNA-induced silencing complex, HB-EGF heparin-binding EGF-like growth factor, EGFR epidermal growth factor receptor, GPNMB transmembrane glycoprotein NMB, BLK B lymphocyte kinase, LRRK2 leucine-rich repeat kinase 2, HIF1α hypoxia-inducible factor 1-alpha, vascular endothelial growth factor VEGF, iNOS inducible nitric oxide synthase, IGF-2 insulin-like growth factor 2, RNP ribonucleoprotein

An interesting study has suggested a lncRNA candidate named LINC00993, which is both considerably deregulated in TNBC and associated with the ER and ANKRD30A gene expression [110]. ANKRD30A (also known as NY-BR-1 or B726P) encodes a DNA-binding transcription factor previously detected in well-differentiated ER-positive and HER2-negative BC tumors [111]. Also, ANKRD30A has been identified as a BC antigen in disseminated tumor cells (DTCs), and is currently one of the most used DTC biomarkers, and a potential target for BC immunotherapy, so the correlated expression between lncRNA LINC00993 and ANKRD30A gene has supported strong evidence that ANKRD30A gene expression may be epigenetic-target of the lncRNA LINC00993; however, more studies are needed in this regard [112–114]. Some lncRNAs have been proposed as competitive endogenous RNA (ceRNA) for short non-coding RNA (miRNAs). LincRNA-RoR (regulator of reprogramming) is upregulated in pluripotent cells (shown in Fig. 2a) [115], where it functions as ceRNA for miR-145, thereby protecting pluripotency factors from miR-mediated silencing, leading to loss of mature miR-145 expression [116]. Recently, Eades et al. found that in TNBC, loss of miR-145 promotes tumor cell invasion. This is mediated through ARF6 overexpression, a protein implicated in tumor invasion through disturbance of cell-cell adhesion by endocytose E-cadherin. In this case, lincRNA-RoR generates a competitive inhibition of miR-145, which alters ARF6 expression. The authors also reported an overexpression of lincRNA-RoR in lymph node positive tumors of TNBC patients and reported the first ceRNA network in human cancer (shown in Fig. 2a) [117, 118].

The expression of other lncRNAs, like HOTAIR, has been shown to enhance the growth and metastasis in xenograft mammary tumors [97]. Wang et al. showed that HOTAIR expression is closely correlated with primary TNBC tumor tissues and demonstrated that HOTAIR expression is transcriptionally repressed by the combined treatment of lapatinib plus imatinib, the first inhibiting EGFR and ErbB2/HER2, and the second a c-ABL inhibitor through β-catenin-binding sites LEF1/TCF4 [119]. In another study, Jin et al. showed that metastasis-associated lung adenocarcinoma transcript 1 (MALAT1) lncRNA exerts its oncogenic activity by interacting with miR-1. MALAT1 was found upregulated in TNBC tissues and is associated to tumor growth and metastasis, as well as poor overall survival. Downregulation of MALAT1 increased the expression of microRNA-1 (miR-1), while overexpression of miR-1 decreased MALAT1 expression. In this sense, MALAT1 exerted its function through the miR-1/slug axis and therefore MALAT1 may be a target for TNBC therapy (shown in Fig. 2b) [120]. Recently, Lin et al. showed that the long intergenic non-coding RNA for kinase activation (LINK-A) is critical for growth factor-induced normoxic signaling pathway by recruiting breast tumor kinase (BRK) activated together with leucine-rich repeat kinase 2 (LRRK2). The latter phosphorylates hypoxia-inducible factor 1-alpha (HIF1α) at Tyr565 and Ser797. The phosphorylation at Tyr565 inhibits hydroxylation at the adjacent Pro564, which prevents HIF1α degradation under normoxic conditions. Ser797 phosphorylation facilitates HIF1α-p300 interaction, leading to activation of HIF1α target genes upon heparin-binding EGF-like growth factor (HB-EGF) stimulation. Importantly, both LINK-A expression and activation of the LINK-A-mediated normoxic HIF1α signaling pathway could serve as a therapeutic strategy in TNBC (shown in Fig. 2c) [121]. Downregulation of lncRNAs has also been shown to be associated with worse clinical outcomes. Such is the case of rhabdomyosarcoma 2-associated transcript (RMST), which functions as an oncogene and whose expression has been correlated to a lower overall survival [79].

## Potential lncRNAs as probable epigenetic biomarkers in TNBC

Nuclear lncRNAs may act as an epigenetic regulator or a guide by recruiting chromatin modification factors to cytogenetic locus, but particularly at gene regulatory/promoter sequences (shown in Fig. 1). As scaffold archetype, nuclear lncRNAs bring together multiple proteins to conform ribonucleoprotein (RNP) complexes. Such lncRNA-RNP complexes can either affect histone modifications or stabilize signaling complexes or nuclear structures [9], as decoy, signaling, and/or guide functional archetypes, as well (Fig. 1).

**Fig. 3:**
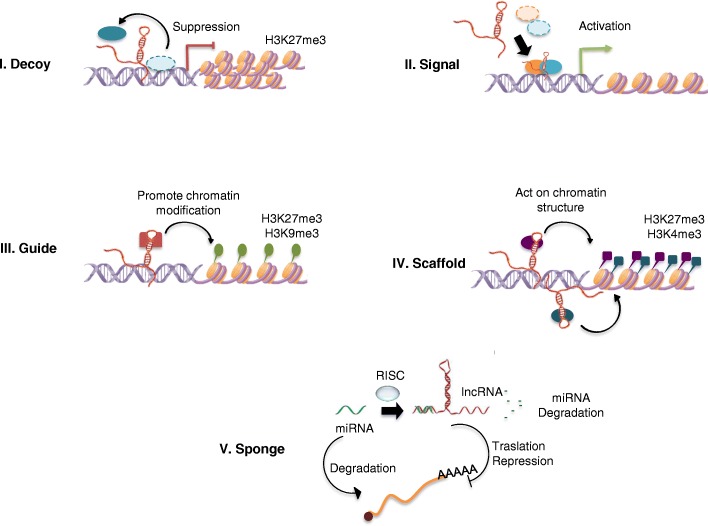
Epigenetic implications of lncRNAs in the development of TNBC. a) MALAT1 regulated by KDM5B and has-miR-448. b) LOC554202 as a host gene of miR-31 (tumor suppressor miRNA), WAVE3 (WAS protein family member 3) KDM5B (lysine-specific demethylase 5B also known as histone demethylase JARID1B), H3K4me3 (trimethylation of lysine 4 on the histone H3 protein subunit), H3K4me1 (monomethylation of lysine 4 on the histone H3 protein subunit), hsa-miR-448 (also known miRNA448), BRCA1/2 (breast cancer 1/2), pRB (retinoblastoma protein), CAV 1 (caveolin 1) HOXA5 (Homeobox protein Hox-A5), SFN (Stratifin), CH3 (methyl group), and RhoA (Ras homolog gene family, member A)

Recently, Rahman et al. have identified lncRNA lnc00673 (ERRLR01) as a marker of overall survival (OS) in BC patients. Specifically, ERRLR01 levels were elevated in TNBC as compared with BC ERα-positive patients. LncRNA ERRLR01 expression levels were also inversely correlated with BC survival for all BC patients, suggesting that ERRLR01 is modulated by hormone signaling in BC [122]. Following this observation, Bamodu et al. showed that metastatic BC cell lines exhibited increased expression levels of lysine-specific demethylase 5B protein (KDM5B) and lncRNA MALAT1, suggesting a functional association. However KDM5B silencing in TNBC cells has been correlated with the upregulation of hsa-miR-448 and led to suppression of MALAT1 expression with a decreased migration, invasion, and clonogenic capacity in vitro, as well as poorer overall survival in vivo (shown in Fig. 3 a) [123]. On the other hand, some miRNAs (microRNAs) that control gene expression by post-transcriptional regulation have been shown to be transcribed as part of host genes. For example, miR-31 is a tumor suppressor-miRNA which is transcribed from the first intron of a host gene LOC554202, on human chromosome 9 [124]. On the other hand, some short non-coding RNA mediate oncogenic processes, such as miR-31, which regulates a group of pro-metastatic target genes, including WAVE3, RhoA, Radexin, and several integrin subunits that regulate key steps in the invasion metastasis cascade [125, 126]. Augoff et al. in 2012 identified a major CpG island upstream of the miR-31 locus, which also spans the first exon of LOC554202, suggesting an epigenetic regulation by methylation of both miR31 and the host gene in basal TNBC compared to luminal BC cell lines (shown in Fig. 3 b) [127].

## Perspectives: lncRNAs as potential therapeutic targets

As we have shown, lncRNAs play several roles in TNBC, but their biological participation is not yet fully understood. Some important advances have been reached, such as the study by Wang et al. which describes different expression patterns of lncRNAs in TNBC vs. non-cancer tissue. We believe that this opens new ideas for functional studies on lncRNAs that have not yet been totally defined as modulators of mRNA coding genes [119]. The lack of complete patterns impedes the development of new TNBC molecular targets, as well as, new-targeted drugs, which could specifically target functional lncRNAs.

However, it would be a fascinating and novel therapeutic strategy. On that recently, Xia et al. designed one oligonucleotide with some chemical modifications which improve its half-life in serum, this molecule antagonizes the function of one tumorigenic lncRNA named ASBEL [128]; in this regard, they have proposed it as a new field of research of potential therapeutic tools for the treatment of TNBC, also named gene therapy.

Notably, lncRNAs could be detected in human bodily fluids, acting as biomarkers. Chen et al. provided useful information for exploring potential therapeutic targets for TNBC [110]. Recently, studies have demonstrated that lncRNA expression could be regulated by conventional chemotherapy agents like tyrosine kinase receptors (TKRs) and non-TKRs by targeting multiple genes at the same time through unknown mechanisms [120]. More studies that strongly focus on molecular mechanisms are needed in order to improve our understanding of how these FDA-approved chemotherapeutic agents for malignant neoplasms exert regulatory action through epigenetic mechanisms on TNBC.

We also know the existence of lncRNA domains upon chromatin structure, where it plays a critical role in the development and/or progression of TNBC disease. Shen et al. explained that chromosome 1 and 10 are the major domains of dysregulation of lncRNAs and mRNA expression, both regulated by lncRNAs through an unknown mechanism [108]. Our research group suggests co-localization of lncRNAs that dictates oncogenic decisions during the development of aggressive TNBC. Several lncRNAs are implicated on hormonal resistance therapy [100]. While some platforms like Oncotype and MammaPrint help medical staff to better identify which patients will respond to standard chemotherapy and have a better prognosis, here, we take into consideration co-expressed mRNAs/lncRNAs that could identify TNBC patients that could benefit from personalized pharmacological treatments. LncRNAs as biomarkers and their associated genetic-epigenetic and transcriptional mechanisms in co-expression patterns of mRNA coding genes open new insights for gene expression control, and epigenetic events that could explain pathophysiology and/or pharmacological actions for clinical diagnosis, treatment response, and prognosis of TNBC patients.

## Conclusions

Perhaps we are approaching an era of personalized therapies for TNBC patients, as was initially idealized by Lehmann et al. who elucidated the TNBC heterogeneity [54, 129, 130]. These therapies, probably will aim to reduce the risk of recurrence and disease progression, as main TNBC tumors feature, as well as to develop more targeted and reduced toxic therapies for the six specific subtypes, previously described [130]. Theoretically, personalized treatments should improve stratification and timing of health care by utilizing biological information and biomarkers on the level of molecular disease pathways, genetics, proteomics, and metabolomics [131]. In this regard, it is imperative that we improve our understanding of biological processes such as epigenetic changes that occur by lncRNAs [132], considering lncRNA archetypes (shown in Fig. 1) for TNBC to reach that point, as a probable personalized epigenetic therapy. Efforts have been made in genomics to personalize the TNBC treatments that are currently oncological under use. This review has presented additional evidence that lncRNAs may work as diagnostic biomarkers and therapeutic targets in solid tumors, including BC and TNBC. However, their relative expression levels in various subtypes of human BC [133], particularly the TNBC subtype, remain to be determined [134].

## Abbreviations

AC: Adriamycin® and Cytoxan®
ANKRD30A: Ankyrin repeat domain 30A
ARA-lncRNA: Adriamycin resistance associated
ARF6: ADP-ribosylation factor 6
BC: Breast cancer
BCAR4: Breast cancer antiestrogen resistance 4
BLK: B lymphocyte kinase
BRCA1/2: Breast cancer 1/2
BRK: Breast tumor kinase
CCAT2: Colon cancer-associated transcript 2
CDK: Cyclin-dependent kinase
ceRNA: Competitive endogenous RNA
CYTOR: Cytoskeleton regulator RNA
DFS: Disease-free survival
DNMT: DNA methyltransferase
DTCs: Disseminated tumor cells
EGFR: Epidermal growth factor receptor
EMA: European Medicines Agency
ER-α: Estrogen receptor alpha
ES+: Estrogen receptor positive
FDA: Food and Drug Administration
FdCyd: 5-Fluoro-2′-deoxcytidine
FGFR: Fibroblast growth factor receptor
GPNMB: Transmembrane glycoprotein NMB
GR: Glucocorticoid receptor
HB-EGF: Heparin-binding EGF-like growth factor
HDAC6: Histone deacetylase 6
HER2: Human epidermal growth factor receptor 2
HIF1α: Hypoxia-inducible factor 1-alpha
HOTAIR: HOX transcript antisense RNA
IGF-2: Insulin-like growth factor 2
IL1: Interleukin-1
iNOS: Inducible nitric oxide synthase
INPP4B: Inositol polyphosphate 4-phosphatase type II
IV: Intravenous
lincRNA-RoR: Long intergenic non-protein coding RNA regulator of reprogramming
LincRNA-RoR: Regulator of reprogramming
LINK-A: Long intergenic noncoding RNA for kinase activation
lncRNAs: Long noncoding ribonucleic acids
LRRK2: Leucine-rich repeat kinase 2
MALAT1: Metastasis-associated lung adenocarcinoma transcript 1
MAPK: Mitogen-activated protein kinase
miR-1: MicroRNA-1
miR-31: Tumor suppressor miRNA
miRNA: MicroRNA
MPS1: Serine/threonine kinase monopolar spindle 1
mTOR: Mammalian target of rapamycin
NAC: Neoadjuvant chemotherapy
ncRNA: Non-coding RNA
OA: Oral administration
PARP: Poly (ADP-ribose) polymerase
pCR: Pathological complete response
PD-1: Programmed cell death receptor
PDGF-R: Platelet-derived growth factor receptors
PD-L1: Programmed cell death ligand-1
PI3K: Phosphatidylinositol 3-kinase
PR: Progesterone receptor
PRC2: Polycomb repressive complex 2
PTEN: Phosphatase and tensin homolog
RISC: RNA-induced silencing complex
RMST: Rhabdomyosarcoma 2-associated transcript
RNP: Ribonucleoprotein
SMDC: Small molecule drug conjugate
TFs: Transcription factors
THU: Tetrahydrouridine
TKRs: Tyrosine kinase receptors
TNBC: Triple-negative breast cancer
UCA1: Urothelial cancer associated 1 non-protein coding
UTR 3: Untranslated region 3′
VEGF: Vascular endothelial growth factor
VEGFR: Vascular endothelial growth factor receptor
WAVE3: WAS protein family member 3
