# Correction to: Long non-coding RNAs: implications in targeted diagnoses, prognosis, and improved therapeutic strategies in human non- and triple-negative breast cancer

**DOI:** 10.1186/s13148-018-0537-5

**Published:** 2018-08-08

**Authors:** Rubén Rodríguez Bautista, Alette Ortega Gómez, Alfredo Hidalgo Miranda, Alejandro Zentella Dehesa, Cynthia Villarreal-Garza, Federico Ávila-Moreno, Oscar Arrieta

**Affiliations:** 1 Thoracic Oncology Unit and Laboratory of Personalized Medicine, Instituto Nacional de Cancerología (INCan), San Fernando #22, Section XVI, Tlalpan, 14080 Mexico City, Mexico; 2 Biomedical Science Doctorate Program, National Autonomous University of Mexico, Mexico City, Mexico; 3 Cancer Genomics Laboratory, INMEGEN, Mexico City, Mexico; 4 Biochemistry Department, Instituto Nacional de Ciencias Médicas y Nutrición Salvador Zubirán, Mexico D.F, Mexico; 5 Breast Oncology Department, National Cancer Institute of Mexico, Mexico City, Mexico; 6 Lung Diseases And Cancer Epigenomics Laboratory, Biomedicine Research Unit (UBIMED), Facultad de Estudios Superiores (FES) Iztacala, National University Autonomous of México (UNAM), Mexico City, Mexico; 7 Research Unit, National Institute of Respiratory Diseases (INER) “Ismael Cosío Villegas”, Mexico City, Mexico

## Correction

Upon publication of the original article [1], the authors noticed that the Figs. 1, 2 and 3 were incorrectly given. The correct Figs. 1, 2 and 3 are given below.

**Fig. 1:**
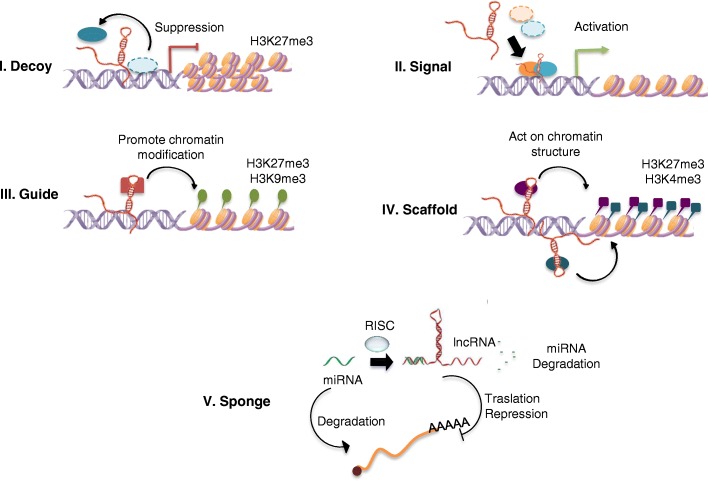
Proposed five functional archetypes for the lncRNA mechanisms. 1. Decoys: lncRNAs can titrate away transcription factors and other proteins away from chromatin, or titrate the protein factors into nuclear subdomains. 2. Signals: lncRNAs expression can faithfully reflect the combinatorial actions of transcription factors (colored ovals) or signaling pathways to indicate gene regulation by space and time. 3. Guides: lncRNAs may recruit chromatin-modifying enzymes to gene-promoter targets, either in Cis (near the genetic region of the lncRNA transcription) or in Trans into distant target genes. 4. Scaffolds: lncRNAs may bring together multiple proteins to conform ribonucleoprotein complexes. The lncRNA-RNP may act on chromatin as illustrated to affect histone code modifications. In other instances, the lncRNA scaffold is structural and stabilizes nuclear structures or signaling complexes 5. Sponge: lncRNAs that by complementarity of bases succeed in matching or sequestering sequences of small non-coding RNAs, such as miRNAs, are controlling bioavailability of miRNAs, vs. lncRNAs themselves, with the functional biological repercussions at cellular or physiological level. RNA-induced silencing complex RISC

**Fig. 2:**
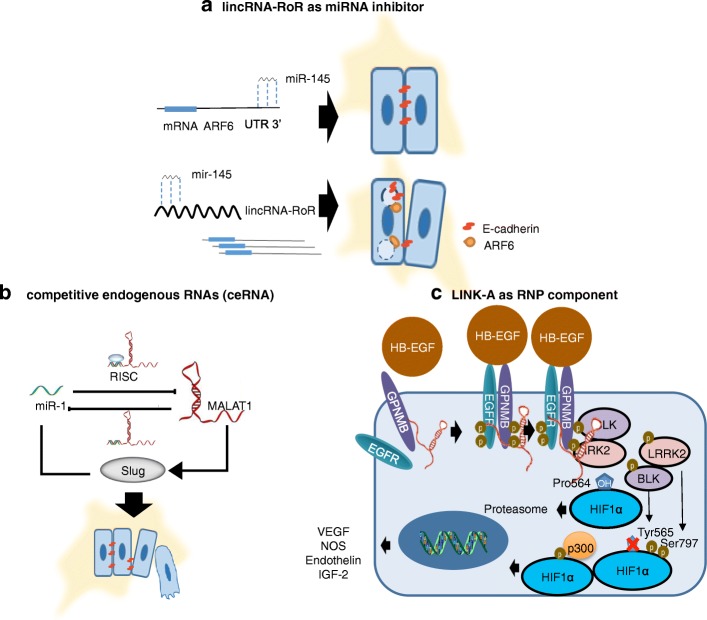
A molecular mechanism model for lncRNAs involved in the tumorigenesis of human TNBC. **a** lincRNA-RoR as a miR-145 inhibitor (oncogene miRNA). **b** MALAT1 as a competitive endogenous RNA of miR-1 (tumor suppressor miRNA). **c** LINK-A as a component of ribonucleoprotein complexes, example shows the regulations of HIF1α pathway. ARF6 ADP-ribosylation factor 6, UTR 3′ untranslated region 3, RISC RNA-induced silencing complex, HB-EGF heparin-binding EGF-like growth factor, EGFR epidermal growth factor receptor, GPNMB transmembrane glycoprotein NMB, BLK B lymphocyte kinase, LRRK2 leucine-rich repeat kinase 2, HIF1α hypoxia-inducible factor 1-alpha, vascular endothelial growth factor VEGF, iNOS inducible nitric oxide synthase, IGF-2 insulin-like growth factor 2, RNP ribonucleoprotein

**Fig. 3:**
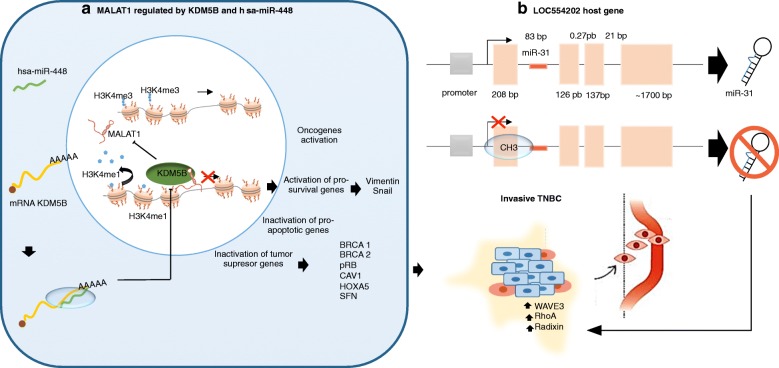
Epigenetic implications of lncRNAs in the development of TNBC. **a** MALAT1 regulated by KDM5B and has-miR-448. **b** LOC554202 as a host gene of miR-31 (tumor suppressor miRNA), WAVE3 (WAS protein family member 3) KDM5B (lysine-specific demethylase 5B also known as histone demethylase JARID1B), H3K4me3 (trimethylation of lysine 4 on the histone H3 protein subunit), H3K4me1 (monomethylation of lysine 4 on the histone H3 protein subunit), hsa-miR-448 (also known miRNA448), BRCA1/2 (breast cancer 1/2), pRB (retinoblastoma protein), CAV 1 (caveolin 1) HOXA5 (Homeobox protein Hox-A5), SFN (Stratifin), CH3 (methyl group), and RhoA (Ras homolog gene family, member A)

The original article has been corrected.
